# The Therapeutic Potential of Four Main Compounds of *Zanthoxylum nitidum* (Roxb.) DC: A Comprehensive Study on Biological Processes, Anti-Inflammatory Effects, and Myocardial Toxicity

**DOI:** 10.3390/ph17040524

**Published:** 2024-04-19

**Authors:** Xiaohan Li, Qi Wang, Ling Liu, Yang Shi, Yang Hong, Wanqing Xu, Henghui Xu, Jing Feng, Minzhen Xie, Yang Li, Baofeng Yang, Yong Zhang

**Affiliations:** 1 Department of Pharmacology, College of Pharmacy, Harbin Medical University, Harbin 150081, China; 17745169030@163.com (X.L.); 15181082096@163.com (L.L.); sy_elisa@163.com (Y.S.); hongyang0672@126.com (Y.H.); xwq8866886@126.com (W.X.); h9606100418@163.com (H.X.); 13069895575@163.com (J.F.); 2 Department of Medicinal Chemistry and Natural Medicinal Chemistry, College of Pharmacy, Harbin Medical University, Harbin 150081, China; wangqiby1987@hotmail.com (Q.W.); xmz446180893@163.com (M.X.); 3 Department of Pharmaceutical Analysis, College of Pharmacy, Harbin Medical University, Harbin 150081, China; liy@hrbmu.edu.cn; 4 Research Unit of Noninfectious Chronic Diseases in Frigid Zone, Chinese Academy of Medical Sciences, 2019 Research Unit 070, Harbin 150081, China; 5 Department of Pharmacology and Therapeutics, Melbourne School of Biomedical Sciences, Faculty of Medicine, Dentistry and Health Sciences University of Melbourne, Melbourne 3010, Australia; 6 Institute of Metabolic Disease, Heilongjiang Academy of Medical Science, Harbin 150086, China

**Keywords:** *Zanthoxylum nitidum* (Roxb.) DC, proteomics, myocardial ion channel, network pharmacology, organ-on-a-chip technology

## Abstract

*Zanthoxylum nitidum* (Roxb.) DC. (*Z. nitidum*) is a traditional Chinese medicinal plant that is indigenous to the southern regions of China. Previous research has provided evidence of the significant anti-inflammatory, antibacterial, and anticancer properties exhibited by *Z. nitidum*. The potential therapeutic effects and cardiac toxicity of *Z. nitidum* remain uncertain. The aim of this research was to investigate the potential therapeutic properties of the four main compounds of *Z. nitidum* in cardiovascular diseases, their impact on the electrical activity of cardiomyocytes, and the underlying mechanism of their anti-inflammatory effects. We selected the four compounds from *Z. nitidum* with a high concentration and specific biological activity: nitidine chloride (NC), chelerythrine chloride (CHE), magnoflorine chloride (MAG), and hesperidin (HE). A proteomic analysis was conducted on the myocardial tissues of beagle dogs following the administration of NC to investigate the role of NC in vivo and the associated biological processes. A bioinformatic analysis was used to predict the in vivo biological processes that MAG, CHE, and HE were involved in. Molecular docking was used to simulate the binding between compounds and their targets. The effect of the compounds on ion channels in cardiomyocytes was evaluated through a patch clamp experiment. Organ-on-a-chip (OOC) technology was developed to mimic the physiological conditions of the heart in vivo. Proteomic and bioinformatic analyses demonstrated that the four compounds of *Z. nitidum* are extensively involved in various cardiovascular-related biological pathways. The findings from the patch clamp experiments indicate that NC, CHE, MAG, and HE elicit a distinct activation or inhibition of the I_K1_ and I_Ca-L_ in cardiomyocytes. Finally, the anti-inflammatory effects of the compounds on cardiomyocytes were verified using OOC technology. NC, CHE, MAG, and HE demonstrate anti-inflammatory effects through their specific interactions with prostaglandin-endoperoxide synthase 2 (PTGS2) and significantly influence ion channels in cardiomyocytes. Our study provides a foundation for utilizing NC, CHE, MAG, and HE in the treatment of cardiovascular diseases.

## 1. Introduction

*Zanthoxylum nitidum* (Roxb.) DC. (*Z. nitidum*), also known as Liang Mian Zhen in Chinese, is the dried root of the Rutaceae plant that is found in the southeastern parts of China, India, and Australia [1]. *Z. nitidum* is known for its anti-inflammatory, analgesic, antimicrobial, and anticancer properties. As a result, *Z. nitidum* is commonly used to treat conditions such as falls and flutter injury, stomach pain, toothache, rheumatic arthralgia, poisonous snake bites, burns, and scalds [2]. NC, CHE, MAG, and HE are abundant and biologically active compounds in *Z. nitidum* [3,4]. Multiple studies have demonstrated the anticancer, anti-inflammatory, antileukemia, and antihuman immunodeficiency virus properties of NC [5,6,7]. Although NC has been shown to induce cytotoxicity through its interaction with topoisomerase I and II, there is also a body of research indicating that it impedes cell proliferation, triggers apoptosis, disrupts the cell cycle, and inhibits DNA ligase activity in cancerous cells [8,9]. As a benzophenone alkaloid, CHE exhibits notable anti-cell proliferation and apoptosis activities. Numerous studies have demonstrated that CHE has diverse antibacterial and antifungal effects [10]. CHE has strong anti-tumor properties against a variety of gastric cancer cell lines by inducing necroptosis [11]. MAG has been shown to help protect the cardiovascular system, regulate immune function, modulate antioxidant properties, reduce inflammation, and lower blood pressure [12,13]. Preclinical studies and clinical trials have provided evidence of the therapeutic effects of HE in a range of diseases, including neurological and psychiatric disorders, as well as cardiovascular diseases. These effects are attributed to the pharmacological mechanism of action of HE, which includes anti-inflammatory, antioxidant, lipid-lowering, and insulin-sensitizing effects [14,15]. Although the anti-inflammatory effects of NC, CHE, MAG, and HE have been reported [16,17,18,19], whether they have anti-inflammatory effects in the heart and their specific mechanisms are unclear. Furthermore, the precise roles of the main compounds of *Z. nitidum* in cardiovascular diseases remain unclear. Notably, NC, CHE, MAG, and HE demonstrate robust biological activity and account for a substantial proportion of *Z. nitidum*. However, their precise functions in the heart and potential cardiotoxic effects remain poorly understood.

The inward recirculating potassium current (I_K1_) plays a crucial role in the maintenance of resting membrane potential and contributes to phase 3 repolarization [20]. Theoretically, the inhibition of I_K1_ can result in membrane depolarization, thereby increasing cellular excitability and automaticity [21]. In addition, the inhibition of I_K1_ can also prolong the action potential duration (APD). Additionally, a reduction in I_K1_ is linked to myocardial ischemia and arrhythmia in myocardial infarction [22]. Previous research has indicated that the activation of the I_K1_ is associated with the persistence of atrial fibrillation and ventricular fibrillation [23]. Intriguingly, prior investigations have indicated that the use of transgenic animals facilitates the induction of Kir2.1 overexpression, resulting in premature ventricular beats, atrioventricular block, atrial fibrillation, and various other arrhythmias [24,25].

L-type calcium channels (LTCCs) are widely distributed within cardiomyocytes, including ventricular muscle, and play a significant role in facilitating increases in intracellular calcium and muscle systole [26]. Many traditional Chinese medicines (TCMs) have meaningful effects on the calcium channels of cardiomyocytes. Wei Lei and his colleagues found that ligustrazine, ferulic acid, senkyunolide I, senkyunolide A, and ligustilide, which were identified as potential LTCC antagonists in Suxiao Jiuxin Pill, a TCM, were widely used as emergency components for treating CHD. The combination of ligustrazine and senkyunolide A showed synergetic calcium antagonism [27]. Sven Baumann et al. reported that wogonin preferentially suppresses T-cell tumor growth through an LTCC-induced Ca^2+^ overload [28].

Organ-on-a-chip (OOC) models are miniature platforms created to mimic dynamic human cell culture and various microenvironments and functions. These devices aim to allow cells to function naturally by incorporating microfluidic flow and 3D tissue reconstruction techniques [29,30]. OOC microfluidic devices, which involve the cultivation of living cells under fluid flow, can accurately mimic organ-level physiology and pathophysiology [31,32]. This approach has the potential to make compound testing more practical by reducing the reliance on animal models, thereby bridging the gap between experimental outcomes and clinical applications in terms of safety and efficacy.

In the present study, we selected four main compounds from *Z. nitidum*, namely, NC, CHE, MAG, and HE. We conducted a first systematic target and pathway analysis of the main compounds in *Z. nitidum* to find the common target PTGS2 and conducted proteomic experiments using NC administered to beagle dogs to clarify the downstream targets and pathways of NC. We also validated the anti-inflammatory effects of the main components in the heart for the first time, and tested the effects on ion channels, thus providing guidance for further secure applications of traditional Chinese medicine and laying a foundation for the transformation of the active ingredients.

## 2. Results

### 2.1. Bioinformatic Analysis of Z. nitidum

**Figure 1:**
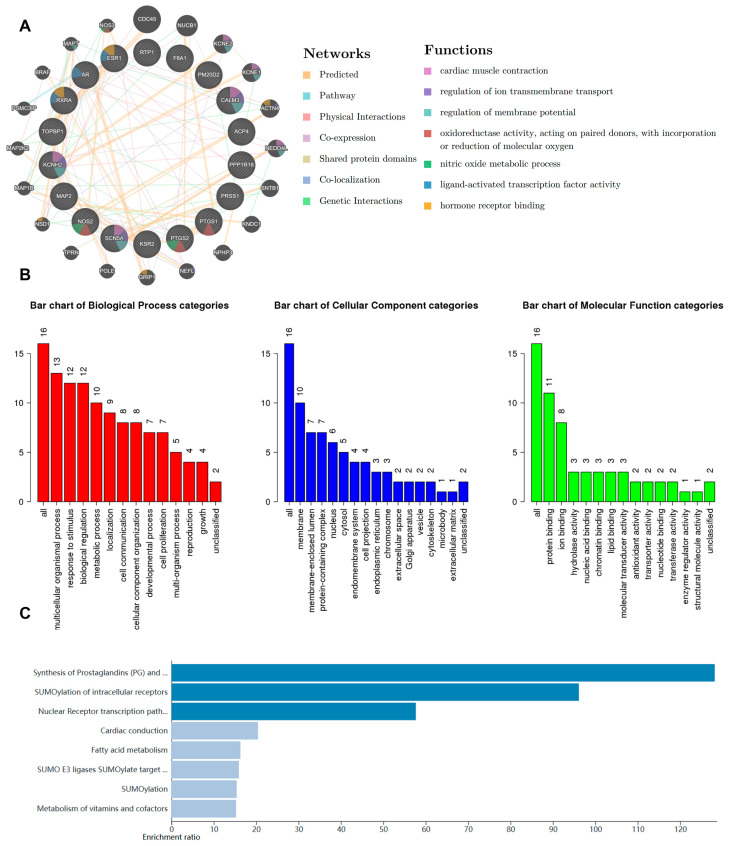
Bioinformatic analysis of *Z. nitidum*. (**A**) PPI network of *Z. nitidum* targets and their potential functions. (**B**) GO map of putative target genes. Biological process, cellular component, and molecular function are included. (**C**) KEGG pathway analysis of *Z. nitidum* target genes.

As a traditional Chinese herbal medicine, *Z. nitidum* contains many compounds with broad applications. We conducted a comprehensive bioinformatic analysis to identify the potential targets of *Z. nitidum*. Using the GeneMANIA platform, we constructed a protein–protein interaction (PPI) network. Our findings indicated a close association between *Z. nitidum* and cardiac physiological processes, particularly the electrical activity of cardiomyocytes (Figure 1A). GO functional analysis and KEGG pathway enrichment analysis were performed on these genes using a bioinformatic tool. The results indicated that *Z. nitidum* might be involved in anti-inflammatory and metabolic pathways (Figure 1B,C). As a traditional Chinese medicine, *Z. nitidum* is commonly employed for the treatment of injuries and inflammatory conditions. Based on the bioinformatic analysis, we proposed that *Z. nitidum* has promising potential in the treatment of cardiac inflammation and the modulation of cardiomyocyte electrical activity.

### 2.2. Determination of the Concentrations of NC, CHE, MAG, and HE in Z. nitidum

Numerous investigations have been conducted on the main compounds in *Z. nitidum* and their associated biological activities. Based on the available literature and bioinformatic analysis and projections, four compounds within *Z. nitidum* including nitidine chloride, chelerythrine, magnoflorine, and hesperidin were chosen for further investigation. 

**Figure 2:**
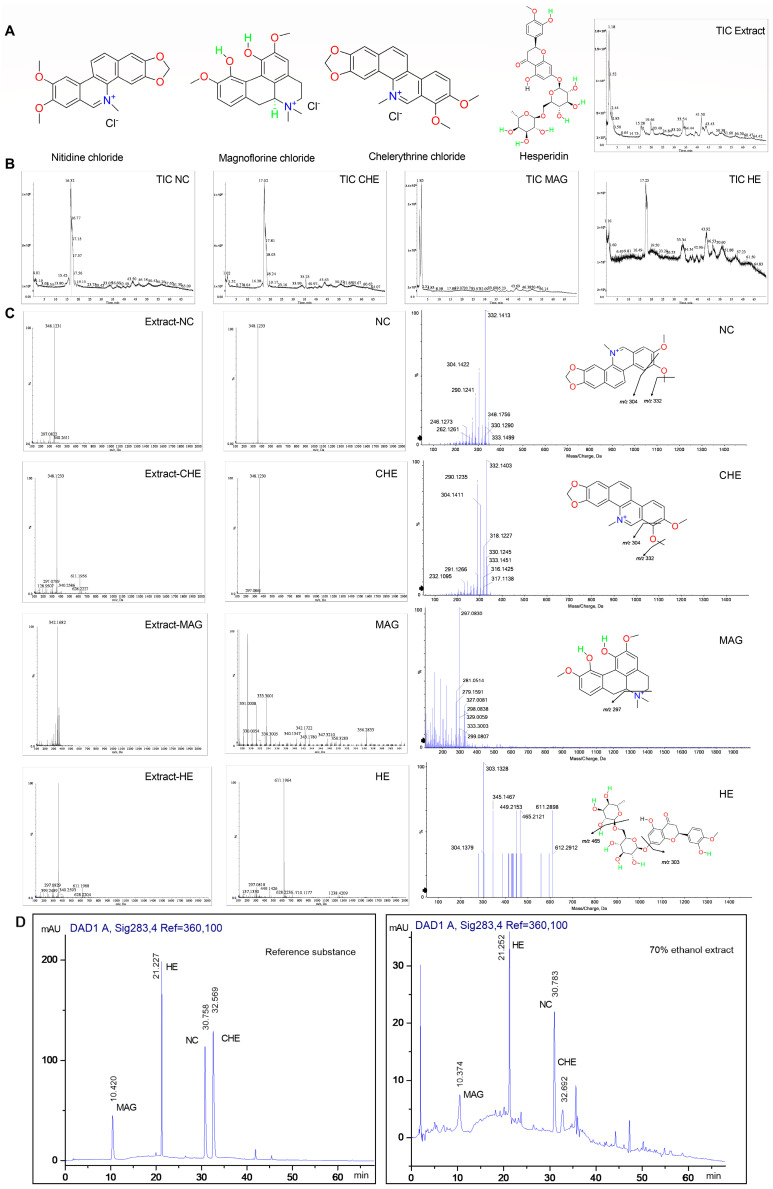
Mass spectrograms and the proposed fragmentation pathways under a positive ion mode. (**A**) Chemical structures of NC, CHE, MAG, and HE. (**B**) Total ion chromatograms (TICs) of four compounds and the extract. of *Z. nitidum*. (**C**) Proposed fragmentation pathways. (**D**) HPLC was used to determine the concentrations of the compounds.

We purchased reference standards with a purity exceeding 99.8% for experimental purposes (Figure 2A). The identification and characterization of compounds in *Z. nitidum* was conducted using UPLC/ESI/Q-TOF-MS in positive ion mode (Figure 2B). From the *m*/*z* value, UV spectrum, retention features, and a comparison with standards, the four compounds were identified from extract of *Z. nitidum*. The parent ions of NC and CHE were at *m*/*z* 348.1231 and 348.1233(C_21_H_18_NO_4_^+^) with a similar fragmentation path. The fragment ions at *m*/*z* 332.1413 (C_20_H_14_NO_4_^+^) and 304.1422 (C_19_H_14_NO_3_^+^) were observed as a result of the elimination of CH_4_ and CO. The mass spectra of MAG showed a molecular ion peak at *m*/*z* 342.1682 (C_20_H_24_NO_4_^+^), and fragment ions at *m*/*z* 297.0830 (C_18_H_17_O_4_^+^) were attributed to the elimination of (CH_3_)_2_NH. This may be an important feature of the aporphine alkaloid fragmentation pathway. HE was identified with the parent ion at *m*/*z* 611.1964 (C_28_H_35_O_15_^+^). The fragment ions at *m*/*z* 465.2121 (C_22_H_25_O_11_^+^) and 303.1328 (C_16_H_15_O_6_^+^) were attributed to the elimination of rhamnoside and rutinose units, respectively (Figure 2C).

Subsequently, we analyzed their content in the 70% ethanol extract of *Z. nitidum* using high-efficiency liquid chromatography (HPLC) (Figure 2D). The selectivity was assessed by analyzing the chromatograms of the extract and reference standards. Linearity calibration curves were constructed using six assays of each reference standard (Table S1). The results of the regression analyses and the correlation coefficients are listed in Table S2. All the compounds showed a good linear relationship in the selected linear range, and the correlation coefficient was between 0.9982 and 0.9998. The limit of quantification (LOQ) was defined as the lowest concentration point of the calibration curve. The limit of detection (LOD) of all compounds was 3 ng/mL (Table S3). In addition, we tested the precision, stability, repeatability, and recovery (Tables S4–S7). After preliminary tests, the sensitivity of the established method met the detection requirements. A quantitative analysis of compounds in *Z. nitidum* was performed by measuring the peak area of each compound. The proportions of the compounds were as follows: 0.54% for NC, 0.29% for CHE, 1.05% for MAG, and 0.91% for HE (Table S8). We found that these four compounds had accounted for a highly substantial proportion of the *Z. nitidum* extract. Subsequently, we investigated the biological functions of these four compounds.

### 2.3. The Function of NC Was Predicted through Proteomics

NC is one of the main components of *Z. nitidum;* thus, we investigated the potential pharmacological functions of NC. 

**Figure 3:**
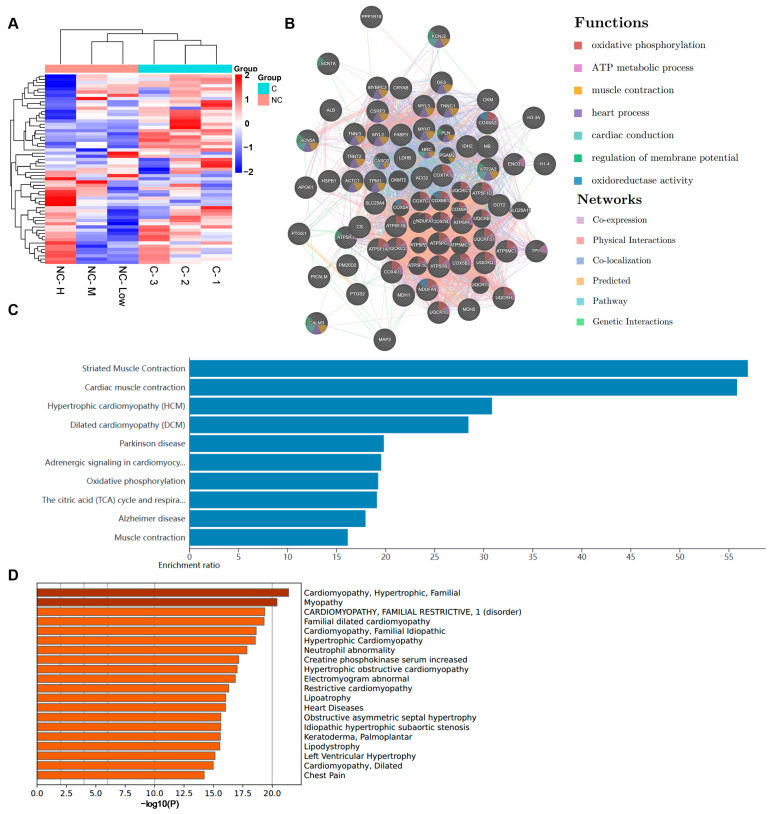
Results of cardiac proteomic analysis in beagles. (**A**) Cluster heatmap of differentially expressed proteins. (**B**) Protein network of NC. (**C**) KEGG pathway enrichment analysis. (**D**) DisGeNET enrichment analysis of NC.

Adult beagles were administered different concentrations of NC (0.375 mg/kg, 0.75 mg/kg, or 1.5 mg/kg). Due to the significant impact of NC on the heart, and the substantial amount of information obtained from the bioinformatic analysis, we conducted a proteomic analysis on heart tissue samples from beagles. The differentially expressed genes were identified and analyzed using a bioinformatic platform. (Figure 3A). We developed a protein–protein interaction (PPI) network for the identified proteins. These proteins were primarily associated with metabolic pathways, muscle contraction, cardiac conduction, and other physiological processes (Figure 3B). We also used bioinformatic tools for a KEGG enrichment analysis (Figure 3C). A component enrichment analysis using the DisGeNET platform showed that NC might be involved in many cardiovascular diseases (Figure 3D).

### 2.4. Prediction of Potential Targets and Pharmacological Characteristics of CHE, MAG, and HE Based on Bioinformatic Analysis

**Figure 4:**
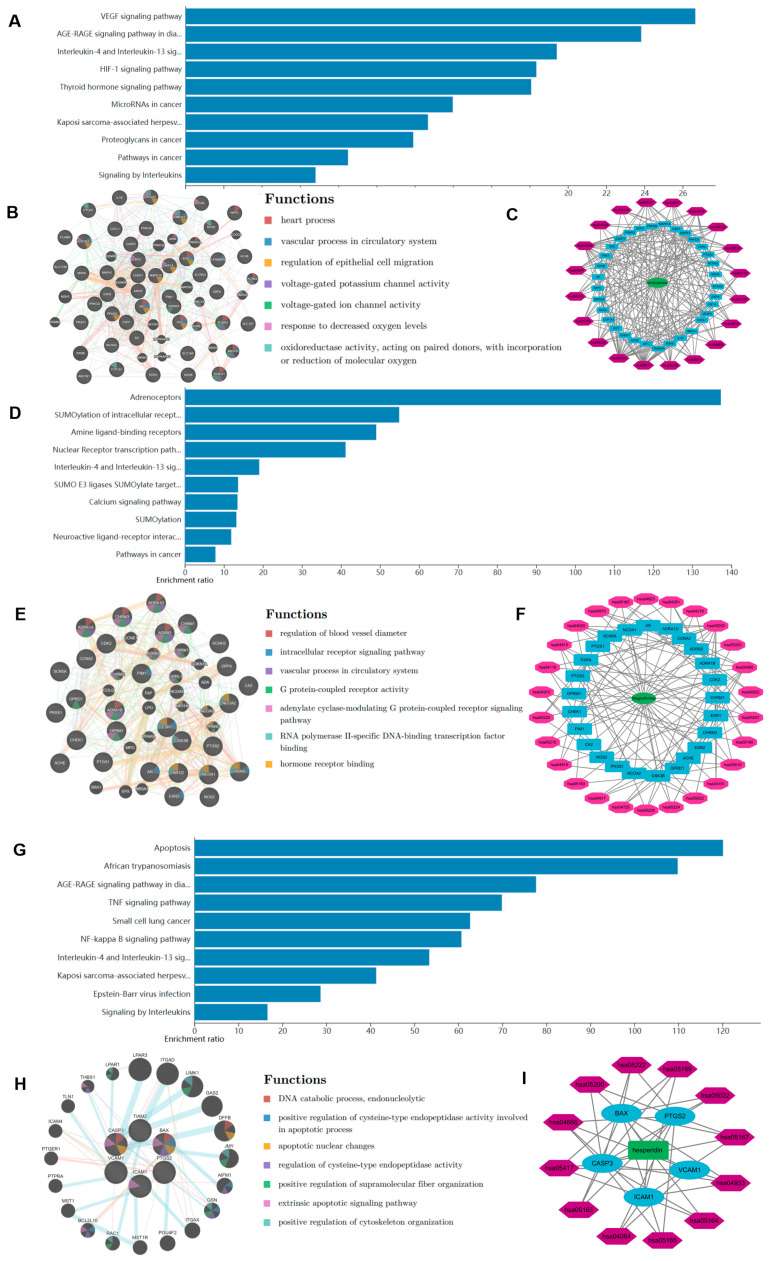
Network pharmacological analysis of CHE, MAG, and HE. (**A**) KEGG pathway analysis of CHE. (**B**) Protein network of CHE. (**C**) Compound–target–pathway map of CHE. (**D**) KEGG pathway analysis of MAG. (**E**) Protein network of MAG. (**F**) Compound–target–pathway map of MAG. (**G**) KEGG pathway analysis of HE. (**H**) Protein network of HE. (**I**) Compound–target–pathway map of HE.

Subsequently, we investigated the physiological processes in which CHE, MAG, and HE may be involved. The targets of these three compounds were preliminarily predicted using the HERB and TCMSP databases. Then, the signaling pathways associated with the targets were analyzed using the WebGestalt platform, and the main biological processes associated with CHE were identified (Figure S1A). The KEGG analysis indicated that CHE plays an important role in numerous physiological processes associated with inflammation and vascular function (Figure 4A). Subsequently, a PPI network was established using the GeneMANIA website (Figure 4B). The results suggest that CHE may be involved in the regulation of the electrical activity of myocardial cells. Certain regions within the PPI network that exhibit high density are referred to as modules; these modules represent potential subnetworks within the PPI network and have distinct biological significance. Hence, to conduct a more precise analysis of the mechanism of action of these compounds, we further delineated their internal modules. Thus, the Metascape platform was used for further analysis. A Metascape visualization was generated to represent the interactive network formed by protein candidates, where the MCODE complexes were colored according to their identities (Figure S1B,C). Subsequently, we generated a compound–target–pathway network diagram using Cytoscape software (v3.9.1), which showed the possible effects of the compounds more intuitively (Figure 4C). Then, we carried out a compound enrichment analysis using the DisGeNET platform (https://www.disgenet.org/), which revealed the diseases in which CHE might be involved in gene regulation. (Figure S1D). TRRUST enrichment analysis was used to identify transcription factors that might be involved in the regulation of CHE targets (Figure S1E). 

We also analyzed MAG and HE in the same way to explore their pharmacological effects. The KEGG analysis revealed that MAG was mainly involved in SUMO modification and might affect myocardial electrophysiological activities (Figure 4D). In addition, we also constructed a PPI network and compound–target–pathway network (Figure 4E,F). Additionally, we further examined additional biological processes associated with MAG (Figure S2A–E). The KEGG enrichment analysis of the PPI network revealed that HE is involved in many inflammatory and apoptotic pathways (Figure 4G–I). Moreover, HE may be involved in many injury-related diseases, especially myocardial ischemia/reperfusion (Figure S3A–C).

### 2.5. Effects of NC, CHE, MAG, and HE on Myocardial Cell Activity

**Figure 5:**
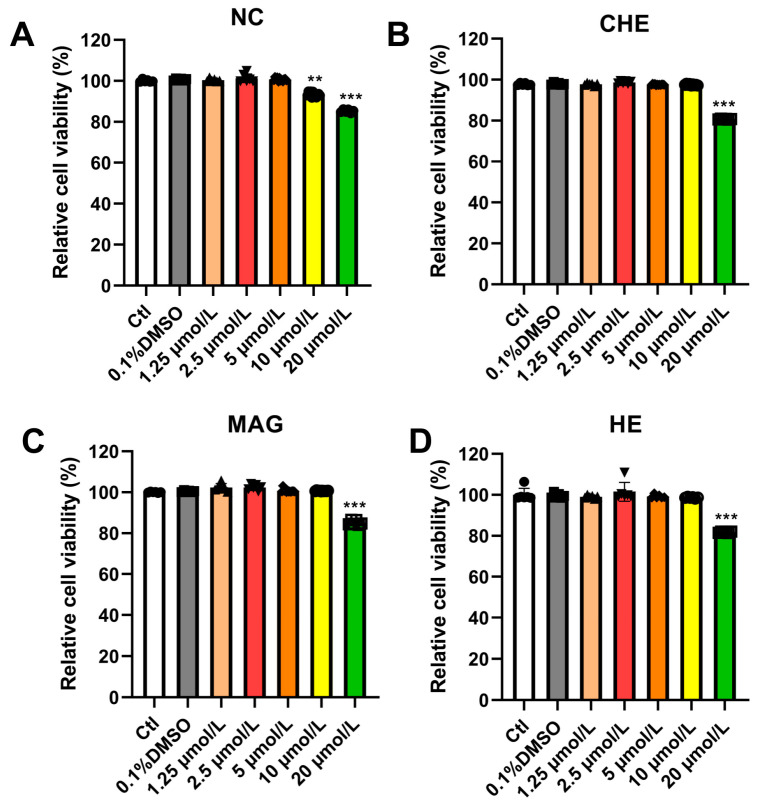
Effects of NC, CHE, MAG, and HE on the viability of myocardial cells. (**A**) CCK-8 was used to determine the viability of NMCMs after treatment with NC for 24 h (*n* = 5). (**B**) CCK-8 was used to determine the cell viability of NMCMs after treatment with CHE for 24 h (*n* = 5). (**C**) Effects of MAG on the viability of myocardial cells (*n* = 5). (**D**) Effects of MAG on the viability of myocardial cells (*n* = 5). The data are presented as the mean ± S.E.M, ** *p* < 0.01, *** *p* < 0.001 vs. the control group.

Subsequently, we assessed the functionality of the individual compounds. First, we performed preliminary investigations to evaluate the impacts of compounds on the activity of myocardial cells and ensure that the functions of the compounds were tested at a suitable concentration (Figure 5A–D). We observed that the compounds had little effect on the activity of myocardial cells at a concentration of 10 μmol/L. Consequently, we continued our investigation based on these findings.

### 2.6. Effects of NC, CHE, MAG, and HE on Myocardial I_K1_

**Figure 6:**
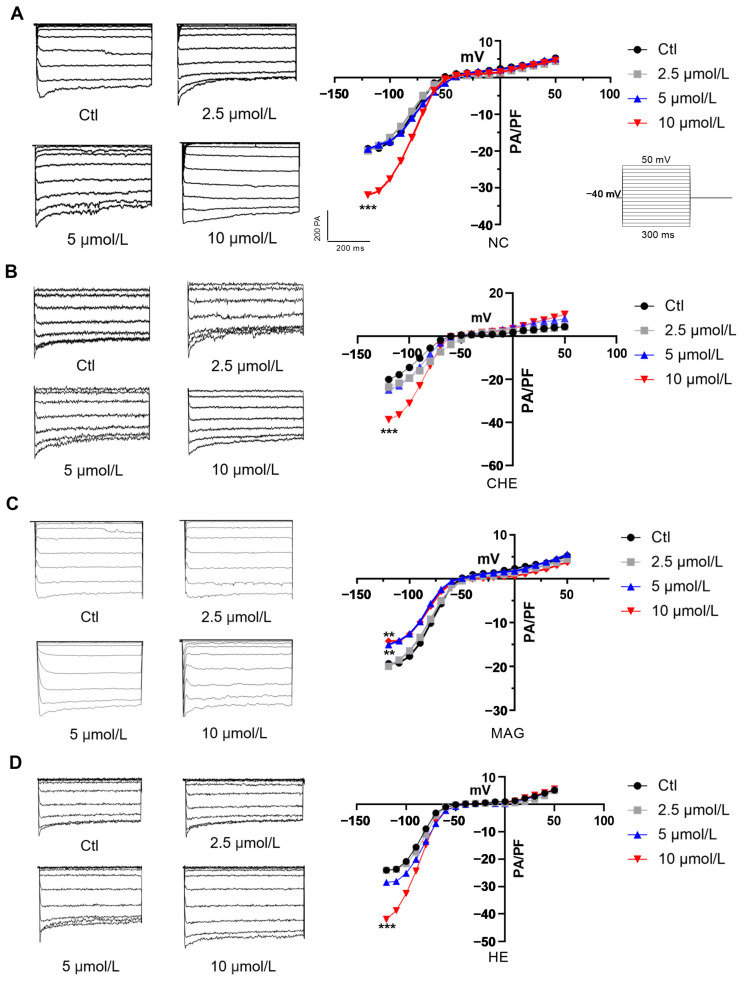
Effects of NC, CHE, MAG, and HE on I_K1_ in the myocardium. (**A**). Representative current traces of I_K1_ and the effects of NC on I_K1_ in the myocardium (*n* = 8). (**B**). Representative current traces of I_K1_ and the effects of CHE on I_K1_ in the myocardium (*n* = 8). (**C**). Representative current traces of I_K1_ and the effects of MAG on I_K1_ in the myocardium (*n* = 8). (**D**). Representative current traces of I_K1_ and the effects of HE on I_K1_ in the myocardium (*n* = 8). The data are presented as the mean ± S.E.M, ** *p* < 0.01, *** *p* < 0.001 vs. the control group.

First, we assessed the effects of the four compounds on myocardial I_K1_. NMCMs were isolated and cultured with compounds at different concentrations (2.5 μmol/L, 5 μmol/L, or 10 μmol/L). The experimental results show that NC had no significant effect on myocardial I_K1_ at 5 μmol/L, while it increased the myocardial I_K1_ at 10 μmol/L, which could induce atrial fibrillation and ventricular fibrillation in cardiomyocytes (Figure 6A). Interestingly, a decrease in the myocardial I_K1_ in myocardial infarction (MI) increased the incidence of arrhythmia. Hence, NC might protect against abnormal cardiomyocyte electrical activity during MI. 

CHE is also one of the main compounds in *Z. nitidum*, and its effect on myocardial I_K1_ was also evaluated. Cardiomyocytes were treated with different concentrations (2.5 μmol/L, 5 μmol/L, or 10 μmol/L) of CHE to determine electrophysiological changes in I_K1_. Similarly to those in the NC group, alterations in myocardial IK1 channels in the 5 μmol/L CHE group were not obvious, while the myocardial I_K1_ dramatically increased with 10 μmol/L CHE treatment (Figure 6B); these results were based on the patch clamp experiment.

Similarly, NMCMs were treated with different concentrations (2.5 μmol/L, 5 μmol/L, or 10 μmol/L) of MAG to evaluate the effects of MAG on myocardial I_K1_. MAG prominently inhibited myocardial I_K1_ at concentrations of both 5 μmol/L and 10 μmol/L. Significantly, the inhibition of myocardial I_K1_ reduced the stability of membrane potential and promoted depolarization (Figure 6C).

Finally, we also examined the effects of HE on myocardial I_K1_. Similarly, primary cardiomyocytes were isolated and treated with different concentrations (2.5 μmol/L, 5 μmol/L, and 10 μmol/L) of HE to determine the effect on myocardial I_K1_. The findings indicated that HE does not have a significant impact on myocardial I_K1_ at a concentration of 5 μmol/L. HE increased the myocardial I_K1_ at 10 μmol/L, which could induce atrial fibrillation or ventricular fibrillation (Figure 6D). Hence, consistent with the effects of NC, HE could also be against aberrant electrical activity during myocardial infarction.

### 2.7. Effects of NC, CHE, MAG, and HE on Myocardial I_Ca-L_

**Figure 7:**
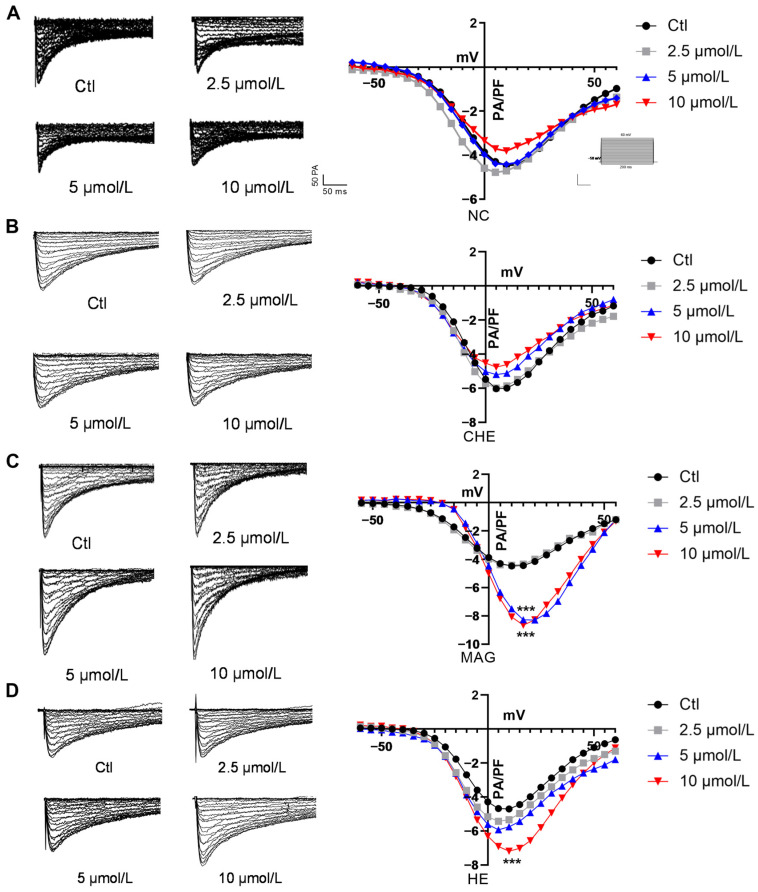
Effects of NC, CHE, MAG, and HE on I_Ca-L_ in the myocardium. Representative current traces of I_K1_. Effects of (**A**) NC, (**B**) CHE, (**C**) MAG, and (**D**) HE on I_Ca-L_ in the myocardium (*n* = 8). The data are presented as the mean ± S.E.M, *** *p* < 0.001 vs. the control group.

When evaluating the alteration in calcium channels, the data show that NC had no significant effect on myocardial I_Ca-L_ at 5 μmol/L. In addition, after treatment with 10 μmol/L, there was a slight decrease in the I_Ca-L_ in cardiomyocytes, with no significant impact on myocardial calcium channels (Figure 7A). These results indicate that the effects of NC on myocardial electronic activity are mainly based on potassium channels.

Subsequently, we also examined the influence of CHE on cardiac calcium currents. Notably, 5 μmol/L CHE slightly inhibited the I_Ca-L_ in cardiomyocytes, whereas 10 μmol/L CHE suppressed the I_Ca-L_ current density (Figure 7B). However, no significant difference was observed in these alterations.

In terms of the impact on I_Ca-L_, MAG significantly increased the I_Ca-L_ in cardiomyocytes with both 5 μmol/L and 10 μmol/L treatment. Consistently, the difference between the two concentrations was not obvious (Figure 7C). Additionally, we noted a depolarization shift in the calcium current following treatment with MAG. This depolarizing shift induced by MAG suggests a more intricate regulation of I_Ca-L_. Above all, the data show that MAG possesses proarrhythmic pharmacological properties.

Regarding calcium currents, 5 μmol/L HE had no meaningful impact on I_Ca-L_, but 10 μmol/L HE increased the peak current density of I_Ca-L_ (Figure 7D). Taken together, these results indicate that HE, to some extent, plays a specific role in promoting arrhythmia.

### 2.8. Predictions of NC, CHE, MAG, and HE Targets and Their Anti-Inflammatory Effects on the Heart

**Figure 8:**
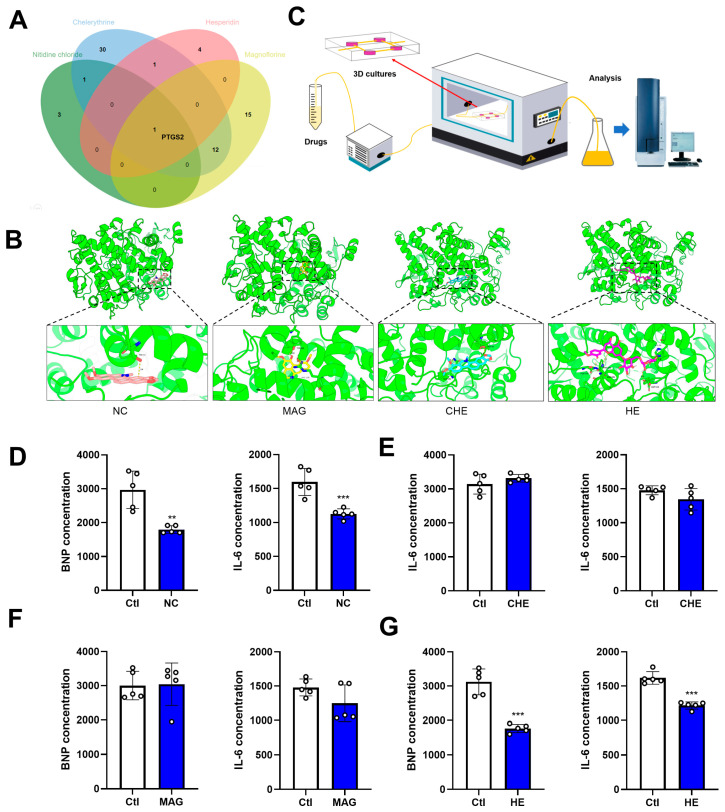
Verification of the targets of the compounds and their anti-inflammatory effects on the heart. (**A**) Venn diagram showing the common targets of the compounds. (**B**) Molecular docking between compounds and PTGS2. (**C**) OOC pattern diagram. (**D**) IL-6 and BNP levels in heart-on-a-chip model treated with NC (*n* = 5). (**E**) IL-6 and BNP levels in heart-on-a-chip model treated with CHE (*n* = 5). (**F**) IL-6 and BNP levels in heart-on-a-chip model treated with MAG (*n* = 5). (**G**) IL-6 and BNP levels in heart-on-a-chip model treated with HE (*n* = 5). The data are presented as the mean ± S.E.M, ** *p* < 0.01, *** *p* < 0.001 vs. the control group.

As NC, CHE, MAG, and HE are the main contents in the bioactive components in *Z. nitidum*, we investigated their common targets and found that PTGS2 is a common target, as was predicted using bioinformatic tools (Figure 8A). The anti-inflammatory impact of *Z. nitidum* might be a result of its influence on the PTGS2 pathway. We utilized molecular docking technology to predict the direct binding of each compound to PTGS2. The results show that the four compounds of *Z. nitidum* could stably bind to PTGS2 (Figure 8B). Given the promising potential of *Z. nitidum* and its bioactive compounds in the cardiovascular system, we evaluated their impact on cardiac anti-inflammation and generated clinical data to support their application in practice. We constructed a heart-on-a-chip model to simulate the heart microenvironment. ELISA results show that NC and HE significantly reduce the levels of BNP and IL-6 in an organic chip medium after 10 μmol/L treatment, which indicates that NC and HE have potential anti-inflammatory effects. Moreover, MAG and CHE had no noticeable effect on the levels of BNP or IL-6 (Figure 8C–G).

For a more in-depth understanding of the pharmacologic effects of *Z. nitidum*, we then assessed the biological activity of the extracts. First, we tested the cytotoxicity of the extracts to ensure that the biological activity of the extracts was tested at the appropriate concentration. We found that the extract did not affect cardiomyocyte activity at a final concentration of 25 mg/L (Figure S4A). We then examined the biological activity of the extract at 25 mg/L. We tested the anti-inflammatory effect of the *Z. nitidum* extract. The results of ELISA show that the extract reduced the levels of IL-6 and BNP in the heart-on-a-chip model, suggesting that the extract could have anti-inflammatory effects (Figure S4B). The results of patch clamp experiments show that *Z. nitidum* extract significantly activated I_K1_ channels in cardiomyocytes (Figure S4C). In addition, we found that the extract inhibited the activity of calcium channels (Figure S4D). 

## 3. Discussion

NC, CHE, MAG and HE are compounds with high concentrations and biological activity in *Z. nitidum*. Recently, Mingbo Jia et al. reported that NC suppresses glioblastoma by inhibiting the epithelial–mesenchymal transition through the regulation of the JAK2/STAT3 signaling pathway. Interestingly, Mingwei Zhu et al. discovered the inhibitory effects of CHE on the progression of glioblastoma. Their research demonstrated that CHE effectively suppresses the TGFB1-ERK1/2/Smad2/3-Snail/ZEB1 signaling pathway, thereby impeding the development of glioblastoma [33]. MAG significantly inhibits the proliferation of prostate cancer cells by altering metabolic biomarkers to disrupt the growth and proliferation of prostate cancer cells [34]. Pankti Patel revealed that HE protected against DMBA-induced breast cancer in female rats by reducing the Ki67 expression [35]. All four compounds in *Z. nitidum* have been fully exploited for the treatment of cancer, and they are also used to treat diabetic [36], inflammatory [7], and neurodegenerative diseases [37]. Four high-content and therapeutically promising compounds in *Z. nitidum* were selected, and their concentrations were quantified using liquid chromatography. Furthermore, in this study, we assessed the impacts of these compounds on cardiac function through proteomics, OOC technology, molecular docking, and patch clamp methods. 

Generally, cardiac arrhythmias frequently occur in individuals with diverse cardiovascular abnormalities, such as MI, coronary atherosclerosis, congestive heart failure, hyperlipidemia, diabetes mellitus, and hypertension [22]. Therefore, cardiac arrhythmia is a common complication that accompanies cardiovascular disease. Disordered cardiomyocyte electrophysiological activity is the leading cause of arrhythmias. Ion channels play a pivotal role in maintaining regular cardiac myocyte electrical activity; once stimulated by external signals, the structure and function of ion channels may become incongruous, and various cardiovascular diseases may be elicited. Many studies have shown that I_K1_ is dominant in determining cardiac excitability. The increase in I_K1_ current promotes arrhythmia due to the decreases in the duration of the action potential and cardiac repolarization [38]. Ca^2+^ plays a pivotal role as a secondary messenger in diverse cell types and is indispensable for a broad spectrum of essential cellular processes, including the contraction of cardiac and skeletal muscle. Intracellular free Ca^2+^ is primarily regulated by ion channels, pumps (such as ATPases), exchangers, and Ca^2+^-binding proteins. Haikel Dridi et al. documented irregularities in the regulation of cytosolic Ca^2+^ with heart failure and atrial fibrillation. These two conditions are widely recognized as the most prevalent forms of heart disease and are significant factors in both mortality and morbidity [39]. Fortunately, the patch clamp recording method, which is a valuable tool for investigating the impact of components on ion channels and forecasting their potential pharmacological effects, is often employed in the development of components [40,41].

In this study, we evaluated the effects of four active compounds from *Z. nitidum* on the cardiac ion channels, I_k1_ and I_Ca-L_, which were investigated using the patch clamp technique. The results indicate that NC, CHE, and HE had no effect on I_k1_ at a concentration of 5 μmol/L but increased the I_k1_ current at a concentration of 10 μmol/L, indicating that these compounds induce aberrant cardiomyocyte electrophysiological activity in a concentration-dependent manner. Intriguingly, the compounds mentioned above may induce an increase in the I_k1_ current under MI conditions, which might explain their protective effects in MI. Moreover, MAG had no impact on I_k1_ current, while MAG and HE had substantial effects on I_Ca-L_. Notably, the efficiency of HE is concentration dependent. Surprisingly, CHE, which is a selective protein kinase C inhibitor, decreases the I_Ca-L_ current; this result is consistent with the findings of Chao-Shun Chan et al., which showed that CHE attenuates the arrhythmogenic effects of sodium hydrosulfide hydrate (NaHS) on pulmonary veins and sinoatrial nodes. 

Ion channels are crucial for maintaining the normal electrical activity of myocardial cells. Both pharmaceutical research and advancements in traditional Chinese medicine require an understanding of how compounds affect ion channels at the molecular level. Patch clamp technology offers valuable technical support, expedites drug research and development, and provides a theoretical foundation for clinical drug use [42,43,44]. However, the electrophysiological activities of cells are intricate, and the therapeutic or toxic effects of drugs cannot be solely determined through patch clamp experiments. Thus, further investigating the effects of drugs in animal or pathological models is essential, and this can be a direction for future research.

The development of OOC technology has led to a profound revolution in in vitro cell culture experiments. This innovative approach is a more accurate replication of human physiology and pathophysiology; thus, this approach provides researchers with valuable insights into disease mechanisms [45]. OOC technology shows significant potential in the evaluation of compound effectiveness and toxicity and can be utilized in the field of pharmaceutical research [46]. Our ongoing research on OOC technology is currently limited to predictive purposes, and further validation of the anti-inflammatory effects of drugs in disease models is necessary. The methods for constructing an inflammation model of the heart using OOC technology are still underdeveloped, and we are actively working on refining this model to enhance our understanding of the efficacy of drugs in future investigations. 

## 4. Materials and Methods

### 4.1. Reagent

K-glutamate, KCl, NaCl, MgATP, MgCl_2_, EGTA, CaCl_2_, and HEPES were purchased from Sigma-Aldrich^®^ Co. (Saint Louis, MO, USA). NC, CHE, MAG, and HE (purity 99.8%) were obtained from Pufei De Biotech Co. (Chengdu, China). NC, CHE, MAG, and HE were dissolved in normal saline following their dissolution in dimethyl sulfoxide (DMSO) (Sigma Aldrich^®^ Co., Saint Louis, MO, USA). The DMSO content in the final medium was maintained below 0.1%.

### 4.2. Zanthoxylum nitidum (Roxb.) DC. Ethanol Extract Preparation

*Z. nitidum* decoction pieces (21.4 kg) were soaked in 70% ethanol (214 L) 10 times for 60 min and then heated at 85–90 °C for reflux extraction for 1 h. The residue was filtered through a 200-mesh screen, and the filtrate was collected. The residue was left for the second extraction. After the first extraction, 10 volumes of 70% ethanol (214 L) were added to the residue, which was heated and refluxed at 85–90 °C for 1 h. Then, residue was filtered through a 200-mesh screen, and the filtrate was collected. After the second extraction, 10 volumes of 70% ethanol (214 L) were added to the residue, which was heated and refluxed at 89–90 °C for 1 h. The residue was filtered through a 200-mesh screen, and the filtrate was collected. The filtrate obtained from the three extractions was combined, and the solvent was recovered in a concentration pot under vacuum (the temperature was not higher than 60 °C) to a relative density of 1.08. A total of 14.48 kg of concentrated extract was continuously dried in a vacuum low-temperature dryer (the drying temperature was not higher than 60 °C under vacuum) to obtain 1.28 kg of 70% ethanol extract of *Z. nitidum*. The dried extract was crushed into whole grains, packed in a sealed bag, and placed in a dryer.

### 4.3. High-Performance Liquid Chromatography

An HPLC system (Agilent Technologies 1200 Series, Santa Clara, CA, USA) was utilized. The extracts were dissolved at a concentration of 2 mg/mL and subsequently filtered through a 0.22 µm membrane filter before injection. The separation of sample compounds was achieved using an Eclipse Plus C18 column (5 µm, 250 mm × 4.6 mm), which was maintained at a temperature of 20 °C. The mobile phase comprised acetonitrile (mobile phase B) and water containing 0.20% H_3_PO_4_ and 0.20% C_6_H_15_N (mobile phase A). The flow rate was maintained at 1 mL/min throughout the analysis, and the gradient program was set as follows: 0–10 min, 88% mobile phase A, and 12% mobile phase B; 10–18 min, 88% mobile phase A, and 12% mobile phase B; 18–30 min, 88–77% mobile phase A, and 12–23% mobile phase B; 30–35 min, 77–72% mobile phase A, and 23–28% mobile phase B; 35–48 min, 72–55% mobile phase A, and 28–55% mobile phase B; and 48–70 min, 40–15% mobile phase A, and 60–85% mobile phase B. The injection volume was 4 µL. 

### 4.4. Ultra-Performance Liquid Chromatography with Electrospray Ionization Quadrupole Time of Flight Mass (UPLC/ESI/qTOF-MS)

The examination was conducted using an AB SCIEX Triple TOF 5600 (Waters, Milford, MA, USA). The autosampler operation was managed using the MassLynxTM 4.1 software (Waters, Milford, MA, USA). In the UPLC analysis, an ACQUITY UPLC CSH Phenyl-Hexyl column (2.1 mm × 178,100 mm, 1.7 μm; Waters, Milford, MA, USA) was utilized for chromatography. The mobile phase was a mixture of acetonitrile and water with 0.1% (*v*/*v*) formic acid. The injection volume was 5 μL, the flow rate was 0.4 mL/min, and the column temperature was maintained at 35 °C. The pressure limit was 15,000 psi. The MS full scan range was 150–1200 *m*/*z*, and the production scan range was 80–1000 *m*/*z*. In the quadrupole time of flight mass (qTOF-MS) analysis, high-purity nitrogen (N_2)_ and high-purity argon (Ar) were utilized as desolvation gas and collision gas, respectively. The flow rate of N_2_ was set at 0.8 L/h. The optimized settings included a capillary voltage of 5.5 kV; declustering potential, 80 V; and collision energy, 35 V. The desolvation and source temperatures were maintained at 450 °C and 100 °C, respectively. All data acquisition in positive ion mode and processing were carried out using Analyst TF (V 1.6).

### 4.5. Organ-on-a-Chip Models

OOC models were fabricated through a PDMS chip model. A cell mixture consisting of 10% endothelial cells, 60% fibroblasts, and 30% cardiomyocytes was utilized to fabricate the OOC model. Subsequently, 100 µL of the cell suspension was added to the chip culture chamber containing 60 µL of Matrigel, followed by thorough shaking and allowing it to sit on a clean bench for one minute. The chip was then cultured at 37 °C in a 5% CO_2_ incubator for two days. Following the successful inoculation and cultivation of cells, the chip was assembled to establish a coculture microsystem. The drug-containing culture medium was then connected to the system at a flow rate of 0.6 μL/min, and the heart OOC was placed into the incubator for dynamic culture.

### 4.6. Prediction of Compound-Related Targets

The search for related targets of rheumatoid arthritis sought potential targets of compounds in the HERB, Pharmmapper, and TCMSP databases. According to the chemical similarity and pharmacophore model, the related targets of compounds were predicted. And the selected targets in the above databases were integrated to obtain potential targets.

### 4.7. Construction of a Network Model 

Based on the data acquired from the annotations of prediction websites, a “compound–target–pathway” network model was constructed using CytoScape software (v3.9.1). After establishing the “compound–target–pathway” network, analyzing the relationships among compounds, targets, and pathways through this network was convenient.

### 4.8. Analysis of the Functional and Pathway Enrichment of the Main Compounds

The WebGestalt platform was used for GO and KEGG enrichment analyses. After entering the target, the *p* value was set, and the analysis result was obtained. The biological process (BP), cellular component (CC), and molecular function (MF) terms were analyzed through GO enrichment analysis, and potential pathways were investigated through the KEGG enrichment analysis.

### 4.9. Protein–Protein Interaction Networks

GeneMANIA is an online analysis tool that is used to generate hypotheses about gene function and determine the priority of genes through functional analysis. The GeneMANIA website was used to construct a protein–protein interaction network for the predicted targets.

### 4.10. Analysis Using Metascape

The interactome network formed by all 121 gene candidates from the Brass list was visualized, with the MCODE complexes colored based on their identities. The summary of the enrichment analysis results from TRRUST and DisGeNET showed the diseases that may be related to the target and transcription factors that may play regulatory roles.

### 4.11. Molecular Docking

Molecular docking is used to verify the binding of compounds and targets. ChemBio3D Ultra 14.0 software was used to minimize the 2D structural energy of the main active compounds of *Z. nitidum*. A three-dimensional structure PDB structure of the target was retrieved from the PDB database. In using AutoDock Tools software (v1.3), the compounds and target proteins were transformed into Pdbqt format for docking. Binding energies less than 0 indicate that compounds and proteins can spontaneously bind and interact. The lower the energy, the more stable the molecular conformation. In general, a binding energy ≤ 5.0 kcal/mol indicates good binding [47,48]. 

### 4.12. Primary Culture of Neonatal Mouse Cardiomyocytes (NMCMs)

Cardiomyocytes were isolated from 1- to 3-day-old neonatal Kunming mice that were purchased from the Experimental Animal Center of the 2nd Affiliated Hospital of Harbin Medical University (Harbin, China). Neonatal mice were sterilized with 75% ethanol before starting the experiment. After opening the chest cavity of a mouse to remove the heart, the heart was placed in a Petri dish and rinsed 2–3 times with an appropriate amount of D-Hank’s solution. An appropriate amount of D-Hank’s and trypsin was added at the ratio of 3 mL of D-Hank’s and 2 mL of trypsin per 30 hearts, and the solutions were then left overnight on a shaker at 4 °C. A total of 16.8 mg of type II collagenase was added to 21 mL of DMEM and set aside in a 37 °C water bath. The hearts were removed, and the digestion was terminated by adding DMEM culture containing 10% serum. Then, twice the heart volume of the prepared type II collagenase culture solution was added and placed in a shaking chamber at 37 °C for 10 min at 160 rpm. The supernatant was pipetted into a new centrifuge tube. The prepared type II collagenase culture solution was added, and the above digestion process was repeated until the heart tissue was digested. The solution was collected and centrifuged at 1500 rpm/min for 5 min. The cells were precipitated at the bottom of the centrifuge tube and resuspended in DMEM supplemented with 10% serum. After that, the cells were evenly spread in culture bottles and cultured in a CO_2_ (5%) incubator at a constant temperature (37 °C) and humidity. After 1.5 h, cardiac fibroblasts were attached to the bottom of the culture bottle, while cardiomyocytes were suspended in the culture medium. The supernatant suspension was collected, diluted, dispersed in fresh culture solution, and evenly spread on a culture plate. After 48 h, the cardiomyocytes adhered to the wall, the morphology and density of the cells were observed via microscopy, and the following experiments were carried out.

### 4.13. Electrode Control

A two-step Faraday microelectrode was fabricated and polished on a drawing instrument (Narishige Company, Tokyo, Japan). The resistance of the microelectrode was 3–8 mΩ.

### 4.14. Experimental Instruments

The patch clamp instrument was a Multi Clamp P700B, Axon; the digital-to-analog converter was a DigiData1440A, Axon; the inverted microscope was an IX51, Olympus; the 3D manipulator was an MP-225, Axon; and the microelectrode drawing instrument was a P97 (Sutter Instrument, Novato, CA, USA).

### 4.15. Patch Clamp Technology

The whole-cell patch clamp technique was used to record the ion current of a single cell. A two-step drawing instrument was used to construct the microelectrode. The resistance of the microelectrode was 3–8 mΩ after filling the inner solution of the electrode. The current signal was collected, stored, and analyzed using a Multiclamp700b patch clamp amplifier, a Digidat 1440A digital-to-analog converter, and Clampfit 10.6. Whole-cell membrane clamp experiments were performed at a room temperature of 22–24 °C. Electrodes filled with pipette solution were mounted on a gripper before the start of the experiment. For the recording of Ca^2+^ currents, the bath solution contained the following: TEACl 50, NaCl 95, MgCl_2_ 2, CaCl_2_ 2, HEPES 10, and glucose 10 (adjusted to pH 7.35 with NaOH). The pipette solution contained the following: TEACl 10, CsCl 140, MgCl_2_ 2, HEPES 2, Mg-ATP 1, and EGTA 10 adjusted to pH 7.25 with CsOH. For recording K^+^ currents, the bath solution contained the following: NaCl 132, KCl 4, CaCl_2_ 1.8, MgCl_2_ 1.2, BaCl_2_ 0.2, glucose 10, HEPES 10, and 4-aminopyridine (4-AP) 5 (pH adjusted to 7.4 with NaOH). The pipette solution contained the following: K-glutamate 120, KCl 20, MgCl_2_ 2, HEPES 10, and Mg-ATP 5, adjusted to pH 7.2 with KOH. Cellular currents were recorded using a Multiclamp700b amplifier, and the signals were filtered at 1 kHz and converted to data through an A/D converter. Currents were recorded in voltage clamp mode. The results were compared between groups, and the currents were normalized using the membrane capacitance, and the current densities were expressed as PA/PF.

### 4.16. CCK-8 Assay

NMCMs were inoculated in 96-well plates. NMCMS were treated with different concentrations of the four active compounds of *Z. nitidum* (NC, CHE, MAG, and HE) for 24 h. To sterilize the CCK-8 solution, it was filtered with a 0.2 μm membrane. Subsequently, 10 µL of CCK8 reagent (Abbkine, Wuhan, China) was added to each well of the 96-well plate. Care was taken to not introduce bubbles into the well as they can interfere with the reading. The absorbance (OD) was detected at 450 nm after 1 h of 37 °C incubation [49]. 

### 4.17. Data Processing

The experimental data were analyzed using X ± SPSS software (v19.0) and were subjected to one-way ANOVA and least significant difference (LSD) tests.

### 4.18. Ethics Statement

This study strictly complied with the World Health Organization’s International Guiding Principles for Biomedical Research Involving Animals. All of the experiments were approved by the Animal Care and Use Committee of Harbin Medical University (No. IRB3017621) and conformed to the Guide for the Care and Use of Laboratory Animals published by the US National Institutes of Health (NIH Publication No. 85-23, revised 1996).

## 5. Conclusions

In this study, we selected the four main compounds of *Z. nitidum*, namely, NC, CHE, MAG, and HE, and conducted a bioinformatic analysis to uncover their potential regulatory targets and pathways. Moreover, we identified the shared target PTGS2 and elucidated the anti-inflammatory mechanism of *Z. nitidum*. We simulated and developed an OOC model to mimic the physiological cardiac environment, thereby confirming the anti-inflammatory properties of the compounds in the heart. In addition, we used patch clamps to observe their influence on the electrical behavior of cardiomyocytes. This study not only serves as a benchmark for the use of these compounds for the treatment of arrhythmia and other medical conditions, but also provides a foundation for understanding cardiac toxicity in the context of compound therapy, thereby supporting the broader utilization of pharmaceutical agents.

## Data Availability

Data are contained within the article.

## Supplementary Materials

The following supporting information can be downloaded at https://www.mdpi.com/article/10.3390/ph17040524/s1: Figure S1: Network pharmacological analysis of CHE; Figure S2: Network pharmacological analysis of MAG; Figure S3: Network pharmacological analysis of HE. Figure S4: The biological activity of the extract; Table S1: Concentrations and peak areas of calibration curves; Table S2: Calibration curves; Table S3: Limit of Detection and Limit of Quantification; Table S4: Precision; Table S5: Stability; Table S6: Repeatability; Table S7: Spike-and-recovery experience; Table S8: Content.
