# Correction: Li et al. The Therapeutic Potential of Four Main Compounds of *Zanthoxylum nitidum* (Roxb.) DC: A Comprehensive Study on Biological Processes, Anti-Inflammatory Effects, and Myocardial Toxicity. *Pharmaceuticals* 2024, *17*, 524

**DOI:** 10.3390/ph18101554

**Published:** 2025-10-16

**Authors:** Xiaohan Li, Qi Wang, Ling Liu, Yang Shi, Yang Hong, Wanqing Xu, Henghui Xu, Jing Feng, Minzhen Xie, Yang Li, Baofeng Yang, Yong Zhang

**Affiliations:** 1 Department of Pharmacology, College of Pharmacy, Harbin Medical University, Harbin 150081, China; 17745169030@163.com (X.L.); 15181082096@163.com (L.L.); sy_elisa@163.com (Y.S.); hongyang0672@126.com (Y.H.); xwq8866886@126.com (W.X.); h9606100418@163.com (H.X.); 13069895575@163.com (J.F.); 2 Department of Medicinal Chemistry and Natural Medicinal Chemistry, College of Pharmacy, Harbin Medical University, Harbin 150081, China; wangqiby1987@hotmail.com (Q.W.); xmz446180893@163.com (M.X.); 3 Department of Pharmaceutical Analysis, College of Pharmacy, Harbin Medical University, Harbin 150081, China; liy@hrbmu.edu.cn; 4 Research Unit of Noninfectious Chronic Diseases in Frigid Zone, Chinese Academy of Medical Sciences, 2019 Research Unit 070, Harbin 150081, China; 5 Department of Pharmacology and Therapeutics, Melbourne School of Biomedical Sciences, Faculty of Medicine, Dentistry and Health Sciences University of Melbourne, Melbourne 3010, Australia; 6 Institute of Metabolic Disease, Heilongjiang Academy of Medical Science, Harbin 150086, China

**Error in Figure**

In the original publication [1], there was a mistake in Figure 7: the current traces in Figure 7D for the 5 μmol group were accidentally presented incorrectly. This inaccuracy was due to an oversight during the preparation of the figures. The authors have carefully checked the raw data and corrected the accidentally confusing image. The correct Figure 7 appears below. All authors agree on this correction to our negligence, and we agree with the corrected images presented below. The authors state that the scientific conclusions are unaffected. This correction was approved by the Academic Editor. The original publication has also been updated.

## Figures and Tables

**Figure 7:**
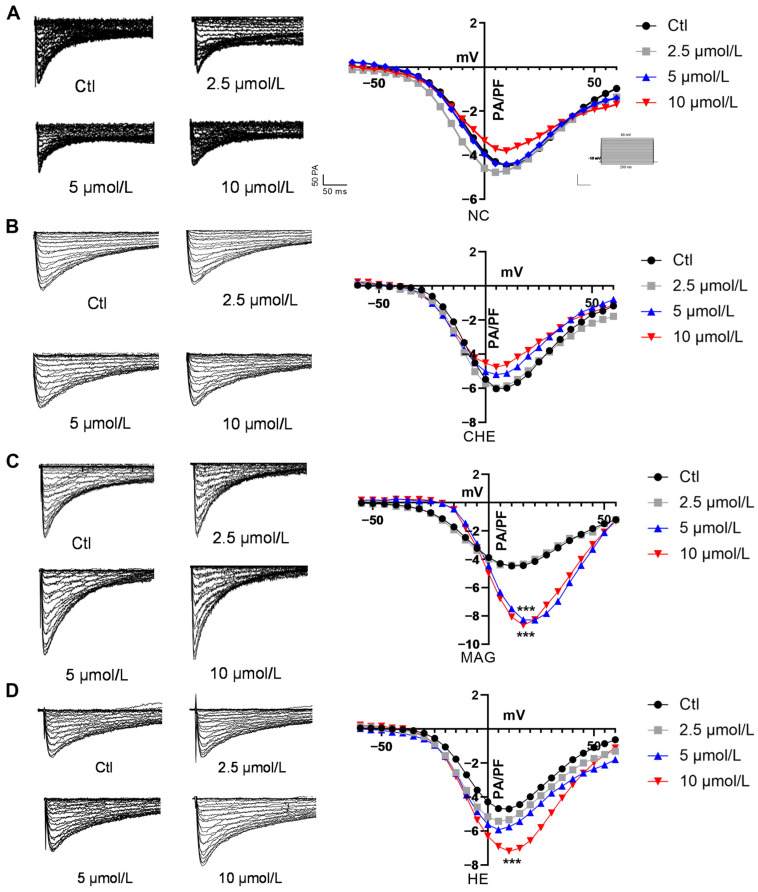
Effects of NC, CHE, MAG, and HE on I_Ca-L_ in the myocardium. Representative current traces of I_K1_. Effects of (**A**) NC, (**B**) CHE, (**C**) MAG, and (**D**) HE on I_Ca-L_ in the myocardium (*n* = 8). The data are presented as the mean ± S.E.M, *** *p* < 0.001 vs. the control group.
