# Supplementary material for: The Therapeutic Potential of Four Main Compounds of Zanthoxylum nitidum (Roxb.) DC: A Comprehensive Study on Biological Processes, Anti-Inflammatory Effects, and Myocardial Toxicity
Source: Pharmaceuticals (Basel). 2024 Apr 19;17(4):524. doi: 10.3390/ph17040524 (PMC11054278; doi:10.3390/ph17040524)
Supplement: Supplementary file 1 [file pharmaceuticals-17-00524-s001.zip › pharmaceuticals-2926929-supplementary.pdf]

Table S1

Concentration and peak area of Calibration curves

μg/mL	MAG Peak area	HE Peak area	NC Peak area	CHE Peak area
1	13.5	14.8	61.3	44.4
10	153.8	145.8	631.4	448.8
20	324	308.7	1344.2	956.1
50	765.5	754.9	3206.6	2245.8
100	1502.6	1431.9	6249	4759.4
200	2845.8	2789.4	12300.3	10272.4

Table S2

Calibration curves

Compound	Calibration Equation	R^2	Linearity Range
MAG	y = 14.225x + 30.891	0.9991	0.01~2μg
HE	y = 13.909x + 24.386	0.9995	0.01~2μg
NC	y = 61.363x + 68.934	0.9998	0.01~2μg
CHE	y = 51.279x - 135.04	0.9982	0.01~2μg

Table S3

Limit of Detection and Limit of Quantification

	LOQ /μg	LOD /μg
MAG	0.01	0.003
HE	0.01	0.003
NC	0.01	0.003
CHE	0.01	0.003

Table S4

Precision

MAG Peak area	RSD (%)	HE Peak area	RSD (%)	NC Peak area	RSD (%)	CHE Peak area	RSD (%)
765.8	2.03	741.5	2.38	3128	0.47	2390.1	0.24
763.4		698.4		3117.8		2285.7	
747.7		701.5		3098.6		2279.7	
731.7		701.5		3106.9		2283.3	
733.6		702.1		3137.1		2317	
755.9		709.8		3129		2299.4	

Table S5

Stability

Time/ h	MAG	RSD (%)	HE	RSD (%)	NC	RSD (%)	CHE	RSD (%)
0	763.7	2.22	745.2	2.11	3150	1.74	2184.4	2.23
1	754.2		751.6		3163.6		2207.8	
2	730.7		732		3163.3		2265	
4	740.5		741.5		3100.8		2328.6	
6	722.4		744.2		3136.5		2313.3	
8	766.5		735.9		3137.5		2268.7	
12	733.8		702.4		3177.6		2315.6	
24	742.6		740.3		3011.3		2319.4	

Table S6

Repeatability

mg/mL	MAG Peak area	Content (%)	Average content (%)	RSD (%)	HE Peak area	Content (%)	Average content (%)	RSD (%)
2.01	356	1.13	1.103	1.99	276	0.91	0.904	0.86
1.96	350.2	1.14			275.4	0.92		
1.98	345.8	1.11			281.5	0.93		
2.04	345.7	1.08			276.6	0.88		
2.03	342.1	1.07			277	0.89		
2.04	337.9	1.05			276.7	0.89		

Repeatability

mg/mL	NC Peak area	Content (%)	Average content (%)	RSD (%)	CHE Peak area	Content (%)	Average content (%)	RSD (%)
2.01	727	0.53	0.535	0.33	133.8	0.91	0.297	0.24
1.96	727.9	0.55			134.6	0.92		
1.98	727.8	0.54			134.3	0.93		
2.04	732.3	0.52			135.8	0.88		
2.03	731.1	0.53			134.5	0.89		
2.04	731	0.52			133.8	0.89		

Table S7

Spike-and-recovery experience

Compound	Sample /mg	Content /μg	Added /μg	Measured /μg	Recovery (%)	Average Recovery (%)	RSD (%)
MAG	2.01	22.13	22	44.09	99.82	99.71	0.12
	2.01	22.13	22	44.03	99.57		
	2.01	22.13	22	44.07	99.73		
HE	2.01	18.29	18	36.12	99.06	99.8	1.02
	2.01	18.29	18	36.17	99.38		
	2.01	18.29	18	36.46	100.98		
NC	2.01	10.88	7	18.08	102.93	103.53	0.5
	2.01	10.88	7	18.15	103.86		
	2.01	10.88	7	18.14	103.81		
CHE	2.01	6.1	7	13.22	101.71	102.28	0.48
	2.01	6.1	7	13.27	102.47		
	2.01	6.1	7	13.28	102.64		

Table S8

Content

Compound		Peak area		Average area	Average content (%)	RSD (%)
MAG	335.3	324.9	333.5	331.2	1.05	1.85
HE	278.8	274.8	275.8	276.4	0.91	0.82
NC	736.5	736	737.5	736.6	0.54	0.11
CHE	135	135.5	136.1	135.5	0.29	0.18

Figure S1

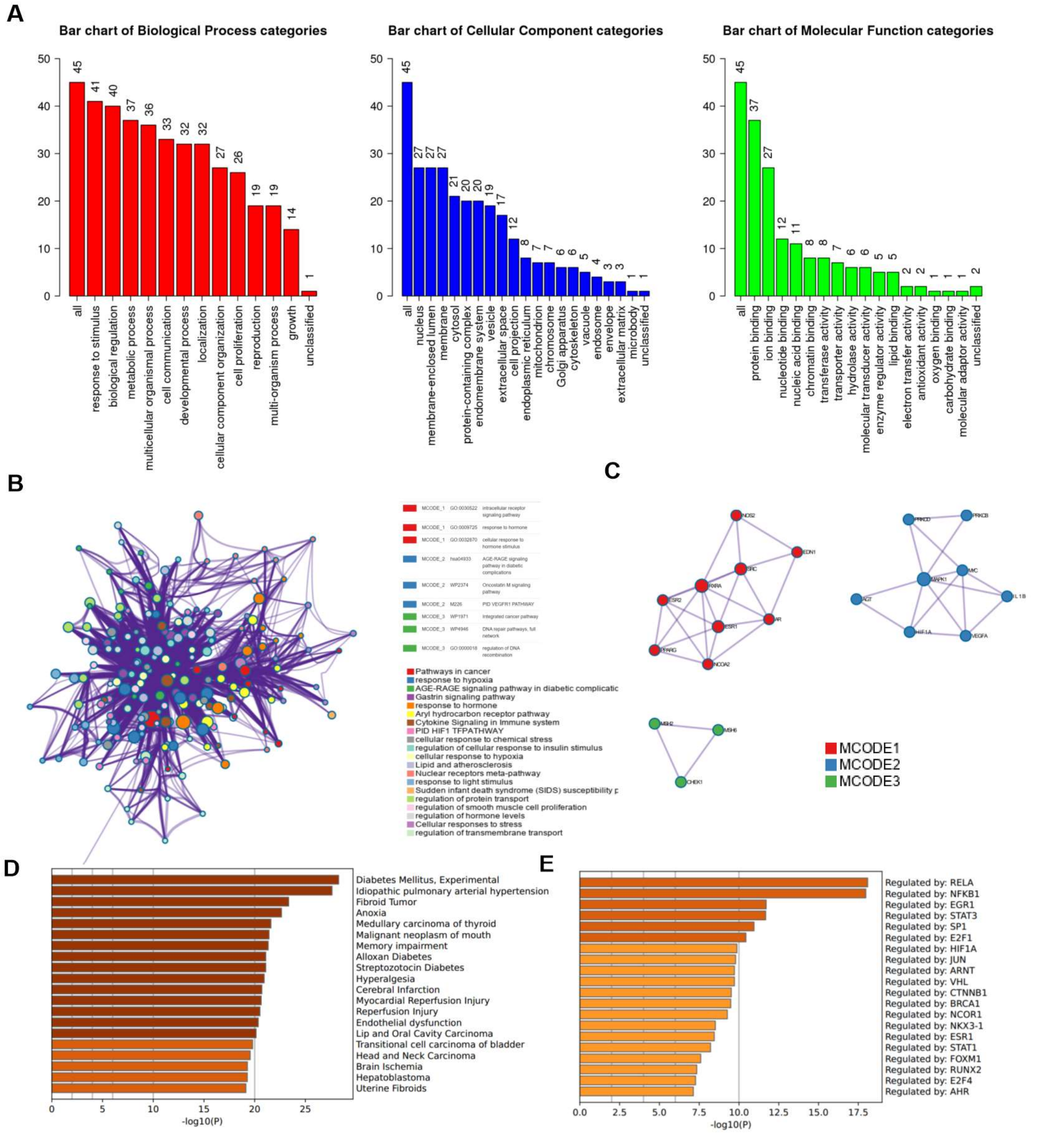

Figure S1. Network pharmacological analysis of CHE. (A) GO function enrichment analysis. (B) Network visualization of the interactive network. (C) MCODE complexes identified in Metascape. (D) Enrichment analysis of CHE on the platform of DisGeNET. (E) Enrichment analysis of CHE on the platform of TRRUST.

Figure S2

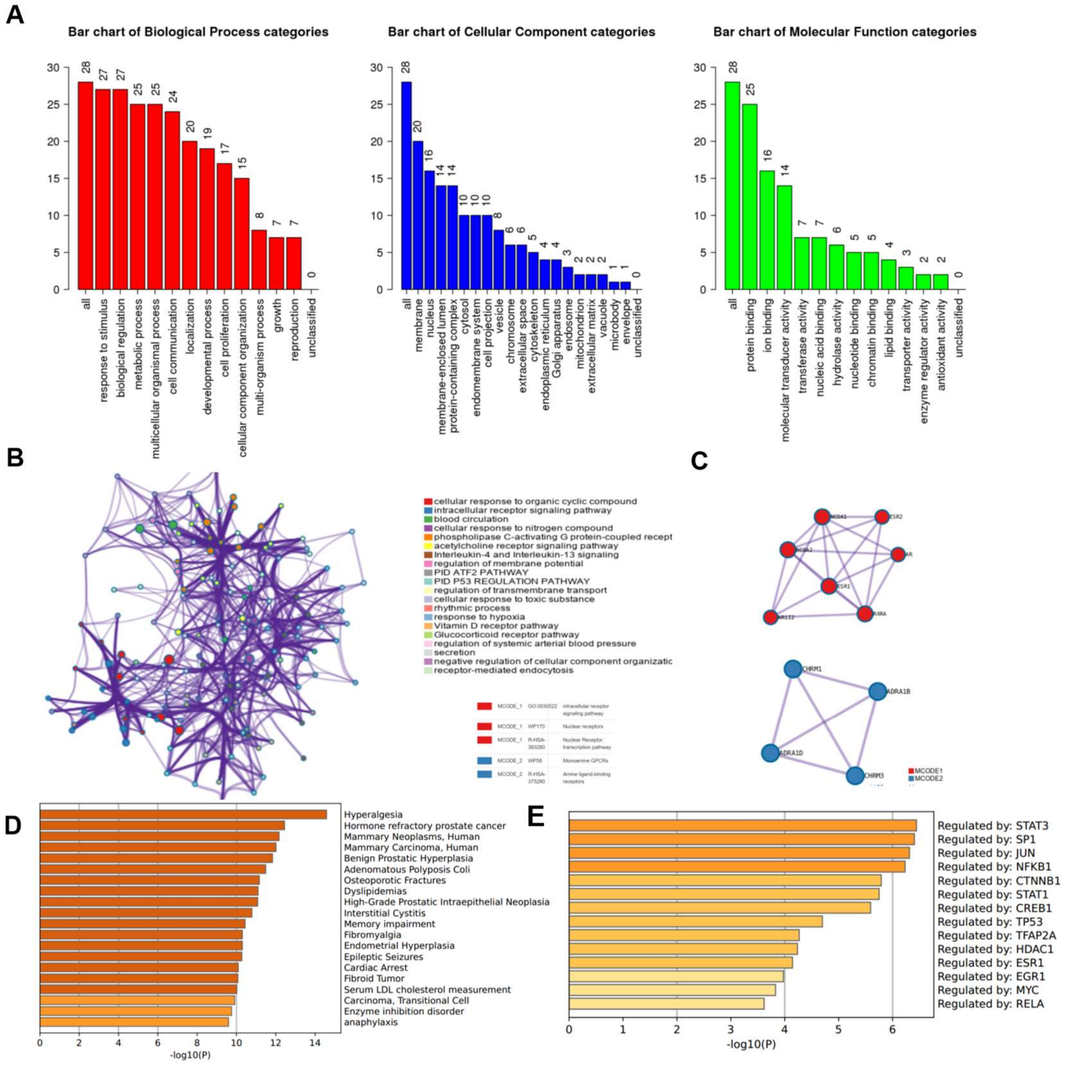

Figure S2. Network pharmacological analysis of MAG. (A) GO function enrichment analysis. (B) Network visualization of the interactive network. (C) MCODE complexes identified in Metascape. (D) Enrichment analysis of MAG on the platform of DisGeNET. (E) Enrichment analysis of CHE on the platform of TRRUST.

Figure S3

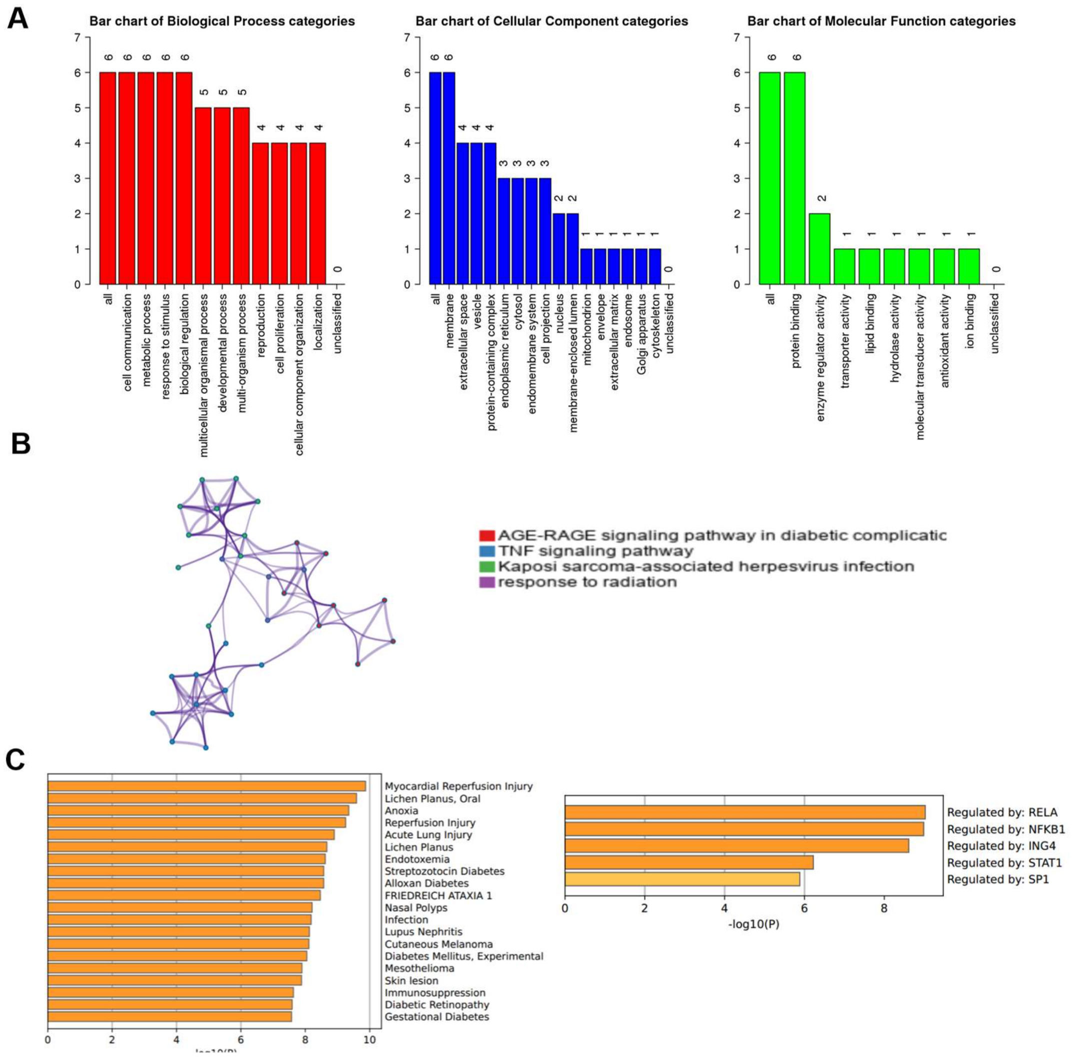

Figure S3. Network pharmacological analysis of HE. (A) GO function enrichment analysis. (B) Network visualization of the interactive network. (C) Enrichment analysis of HE on the platform of DisGeNET and TRRUST.

Figure S4

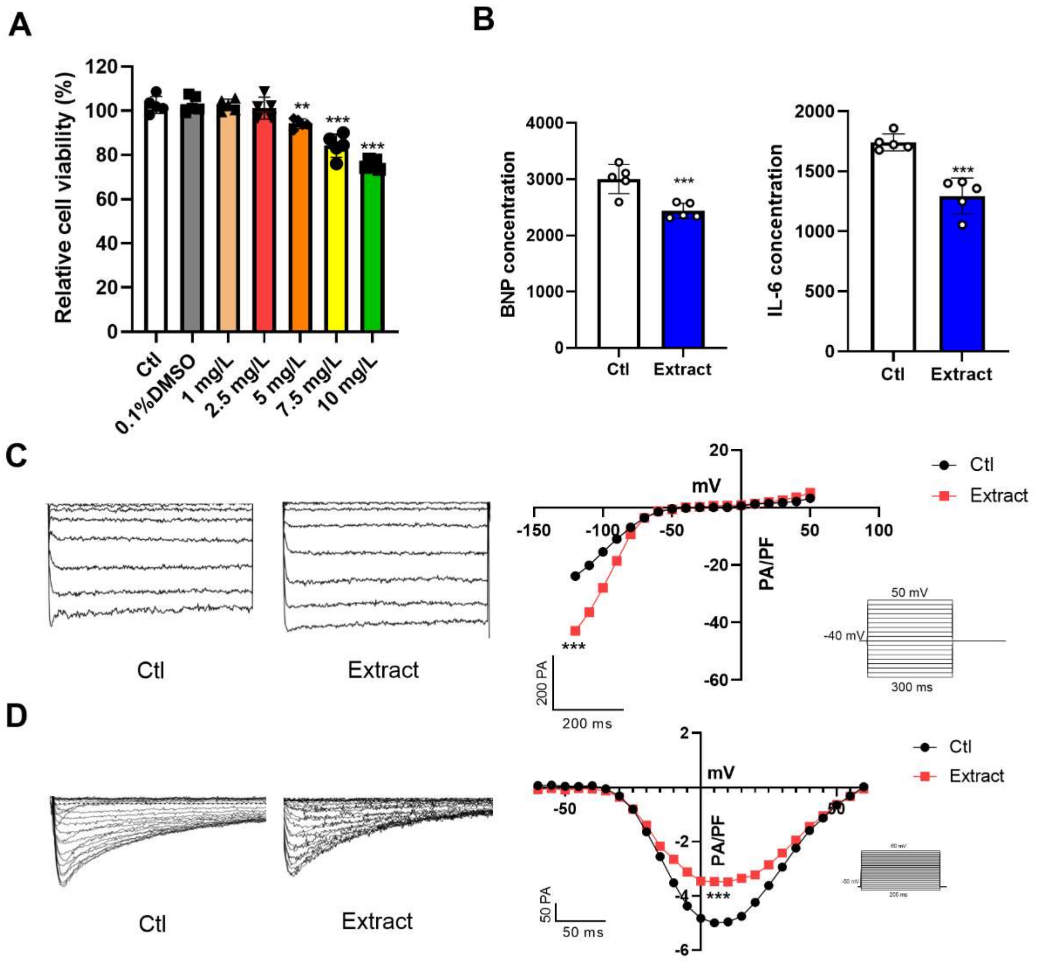

Figure S4. The biological activity of extract. (A) Effect of the extract on the viability of myocardial cells (n = 5). (B) IL-6 and BNP levels in heart-on-a-chip model treated with the extract of *Z. nitidum* (n = 5). (C) Effect of the extract on I_{K1} in the myocardium (n = 8). (D) Effect of the extract on I_{Ca-L} in the myocardium (n = 8). The data are presented as the mean ±S.E.M, **p < 0.01, ***p < 0.001 vs. the control group.
